# Acute Aseptic Meningitis Temporally Associated with Intravenous Polyclonal Immunoglobulin Therapy: A Systematic Review

**DOI:** 10.1007/s12016-024-08989-1

**Published:** 2024-05-13

**Authors:** Elisabetta L.T. De Felice, Gabriel F. Toti, Beatrice Gatti, Renato Gualtieri, Pietro Camozzi, Sebastiano A.G. Lava, Gregorio P. Milani, Giorgio Treglia, Federica Vanoni, Mario G. Bianchetti, Gianmaria F. Bernasconi, Benedetta Terziroli Beretta Piccoli, Camilla Lavagno

**Affiliations:** 1 Family Medicine Institute, Faculty of Biomedical Sciences, Università della Svizzera italiana, Lugano, Switzerland; 2 Department of Anesthesia, Hôpital du Valais, Sion, Switzerland; 3 Pediatric Cardiology Unit, Department of Pediatrics, Centre Hospitalier Universitaire Vaudois, University of Lausanne, Lausanne, Switzerland; 4 Clinical Pharmacology & Therapeutics Group, University College London, London, UK; 5 Pediatric Unit, Fondazione IRCCS Ca’ Granda Ospedale Maggiore Policlinico, Milan, Italy; 6 Department of Clinical Sciences and Community Health, Università degli Studi di Milano, Milan, Italy; 7 Clinic of Nuclear Medicine, Imaging Institute of Southern Switzerland, Ente Ospedaliero Cantonale, Bellinzona, Switzerland; 8 Faculty of Biomedical Sciences, Università della Svizzera italiana, Lugano, Switzerland; 9 Institute of Pediatric of Southern Switzerland, Ente Ospedaliero Cantonale, Bellinzona, Switzerland; 10 Epatocentro Ticino, Lugano, Switzerland; 11 Faculty of Life Sciences & Medicine, King’s College London, King’s College Hospital, London, UK; 12 Pediatric Emergency Department, University Children’s Hospital Zurich, Zurich, Switzerland

**Keywords:** Aseptic meningitis, Autoimmune disorder, Drug-induced meningitis, Intravenous polyclonal human immunoglobulin, Meta-analysis, Systematic review

## Abstract

An acute aseptic meningitis has been occasionally observed on intravenous polyclonal human immunoglobulin therapy. Since case reports cannot be employed to draw inferences about the relationships between immunoglobulin therapy and meningitis, we conducted a systematic review and meta-analysis of the literature. Eligible were cases, case series, and pharmacovigilance studies. We found 71 individually documented cases (36 individuals ≤ 18 years of age) of meningitis. Ninety percent of cases presented ≤ 3 days after initiating immunoglobulin therapy and recovered within ≤ 7 days (with a shorter disease duration in children: ≤ 3 days in 29 (94%) cases). In 22 (31%) instances, the authors noted a link between the onset of meningitis and a rapid intravenous infusion of immunoglobulins. Cerebrospinal fluid analysis revealed a predominantly neutrophilic (*N* = 46, 66%) pleocytosis. Recurrences after re-exposure were observed in eight (*N* = 11%) patients. Eight case series addressed the prevalence of meningitis in 4089 patients treated with immunoglobulins. A pooled prevalence of 0.6% was noted. Finally, pharmacovigilance data revealed that meningitis temporally associated with intravenous immunoglobulin therapy occurred with at least five different products. In conclusion, intravenous immunoglobulin may cause an acute aseptic meningitis. The clinical features remit rapidly after discontinuing the medication.

## Introduction

The term acute aseptic meningitis syndrome denotes a sudden onset condition characterized by symptoms and signs consistent with a meningitis, an elevated white cell count in the cerebrospinal fluid with negative microbiological studies, followed by a typically spontaneous, rapid, and positive course [1, 2]. This syndrome was originally described a century ago by the pediatrician Arvid Wallgren (1883–1973) in individuals with a benign illness resembling viral meningitis [3]. However, its usage has expanded to encompass a wide range of infectious and noninfectious causes [1, 2].

Several drugs, including some nonsteroidal anti-inflammatory agents and antimicrobials like aminopenicillins or sulfonamides, have been temporally associated with aseptic meningitis [1, 2].

Intravenous polyclonal human immunoglobulins are the cornerstone in the treatment of an array of disorders [4]. However, these preparations have also been temporally associated with headaches, fever, flu-like symptoms, nausea, flushing, rash, joint pain, allergic reactions, anemia, and the development of aseptic meningitis [1, 2].

To make informed decisions regarding the administration of intravenous immunoglobulin therapy, healthcare providers need a thorough understanding of the incidence, risk factors, clinical presentation, prevention, management, and outcome of aseptic meningitis temporally associated with this treatment. Therefore, we have conducted a systematic review and meta-analysis of the existing literature on this subject.

## Methods

### Data Sources and Search Strategy

This review was recorded in the Prospective Register of Systematic Reviews with the code PROSPERO CRD42023445798 and was conducted in agreement with the 2020 edition of the Preferred Reporting Items for Systematic Reviews and Meta-Analyses (PRISMA) recommendations [5]. The data sources utilized were Excerpta Medica, the US National Library of Medicine, and Web of Sciences, without any limitation. The search strategy employed the following terms entered in separate pairs: (Intravenous immunoglobulin OR IVIG OR gamma globulin) AND (meningitis OR cerebrospinal inflammation). Relevant articles cited in the retrieved records, reports available in Google Scholar, and reports previously known to the authors were also considered for inclusion. The search was carried out in June 2023 and repeated prior to submission (November 8, 2023).

### Eligibility

Eligible were individually documented cases with the clinical (increased body temperature, nausea, vomiting, headache, neck stiffness, and seizures) and laboratory (white cell pleocytosis in the cerebrospinal fluid) features of acute aseptic meningitis temporally associated with intravenous polyclonal immunoglobulin therapy, negative microbiological studies, and absence of any other explanation for the pleocytosis including meningitis directly induced by an underlying autoimmune disease [6]. From each case of aseptic meningitis associated with a polyclonal immunoglobulin therapy, we extracted following information: (1) demographics; (2) underlying medical condition; (3) dose and type of employed immunoglobulin product; (4) symptoms and signs consistent with a meningitis; (5) signs and symptoms consistent with an anaphylactic reaction (acute onset skin and mucosal lesions together with respiratory, cardiovascular, and intestinal involvement) or a serum sickness (skin rash and joint pain) and impression that meningitis may have been facilitated by a rapid drug administration of polyclonal immunoglobulin [7]; (6) results of cerebrospinal fluid analysis; (7) time latency between initiating immunoglobulin therapy and development of acute meningitis; (8) time required for recovery after discontinuing immunoglobulin therapy and possible sequelae; and (9) recurrent episodes of meningitis and sequelae. Eligible were also case series and pharmacovigilance studies addressing the issue of acute meningitis syndrome temporally associated with polyclonal immunoglobulin therapy.

### Analysis

The nine categories of information extracted from each individual case were rated as 0 or 1, and the reporting quality was graded, according to the sum of these factors, as excellent (≥ 7), good (5 to 6), or satisfactory (4 to 5), according to our standard procedure [8]. Literature search, study selection, data extraction, and comprehensiveness assessment of each retained case were performed in duplicate by two authors. In case of disagreement, a discussion involving a senior author was conducted to resolve any discrepancies. The data were transcribed into a predetermined worksheet by one author, and a second author verified the accuracy of the data entry. Pairwise deletion was used to handle missing data [9]. Categorical data are presented as counts (and sometimes also as percentages). For dichotomous data, the Fisher’s exact test was used, while the Mann-Whitney-Wilcoxon *U* test was employed for ordered categorical variables [10, 11]. Medians and interquartile ranges were used to present continuous data, and the Mann-Whitney-Wilcoxon *U* test was used for their analysis [10]. Two-sided *P* values of less than 0.05 were considered statistically significant. Statistical analysis was conducted using GraphPad Prism, version 10.1.1 for Mac OS X (GraphPad Software, Boston, Massachusetts, USA).

The prevalence of acute aseptic meningitis temporally associated with intravenous polyclonal immunoglobulin therapy was calculated through a proportion meta-analysis. Proportion of acute aseptic meningitis temporally associated with intravenous polyclonal immunoglobulin therapy was calculated using data retrieved from each of the selected studies, and subsequently, a pooled proportion was calculated. We used a random-effects model for the meta-analysis because this statistical method assists in controlling for unobserved heterogeneity among the included studies. The meta-analysis considers that studies may have a different weight in the pooled analysis. We calculated pooled values of the main outcomes including their 95% confidence intervals (95% CI). We have used forest plots for displaying the data. Inconsistency index (*I*^2^-index) was used to assess the statistical heterogeneity among the included studies (*I*^2^-index > 50% is a sign of significant statistical heterogeneity). We used the Egger’s test to assess the presence of a publication bias (*P* value > 0.05 for absence of publication bias). Statistical analyses were performed using an open-source software for meta-analyses (OpenMeta®).

## Results

### Search Results

**Fig. 1:**
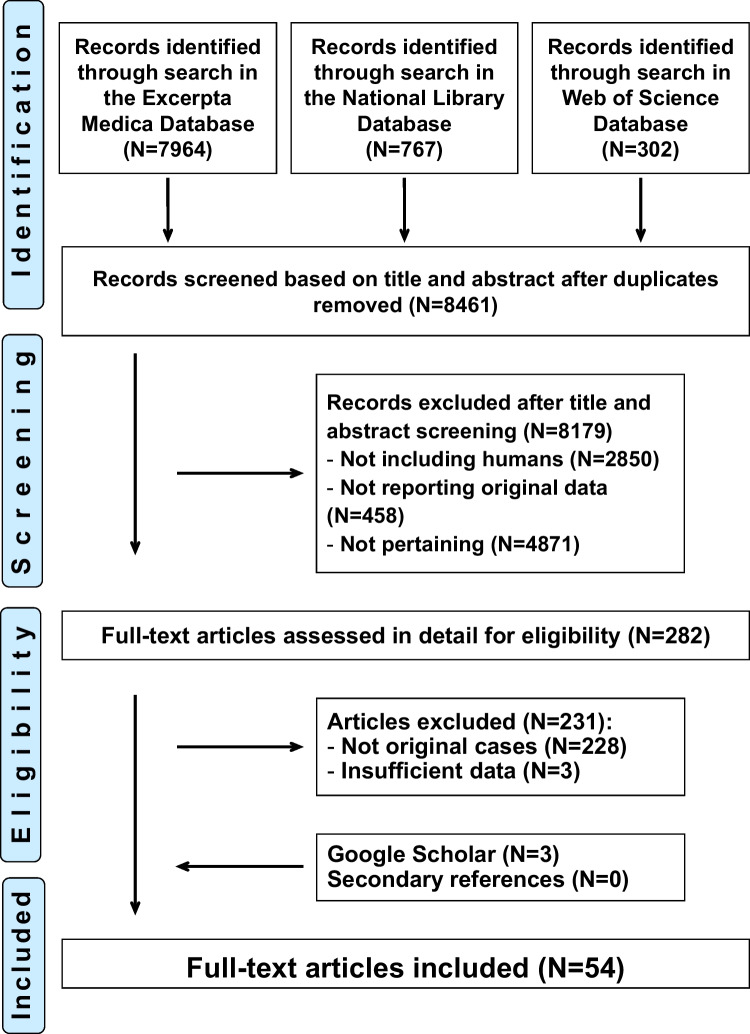
Acute aseptic meningitis temporally associated with intravenous polyclonal immunoglobulin therapy. Flowchart of the literature search

Flowchart of study selection process is depicted in Fig. 1. For the final analysis, we retained 54 communications [12–65] published between 1988 and 2023 in English (*N* = 47), French (*N* = 4), or Spanish (*N* = 3). The articles had been reported from the following continents: 23 from Europe, 18 from America, 11 from Asia, and one each from Oceania and Africa.

Forty-eight reports described 71 patients, who experienced at least one episode of acute meningitis temporally associated with polyclonal intravenous immunoglobulin therapy. Nine reports were case series or pharmacovigilance studies addressing the issue of meningitis associated with polyclonal immunoglobulin therapy.

### Findings

#### Individually Documented Cases of Acute Aseptic Meningitis

The 71 individually documented cases of acute meningitis temporally associated with a polyclonal intravenous immunoglobulin therapy were 36 children and 35 adults (39 females and 32 males). Reporting comprehensiveness was excellent in 60 cases, good in 9 cases, and satisfactory in 2 cases [12–59]. More than 90% of patients were affected by an autoimmune inflammatory disorder, as shown in Table 1.

**Table 1:** Underlying conditions in 71 patients 1.0 to 77 years of age with an acute aseptic meningitis temporally associated with an intravenous polyclonal immunoglobulin therapy

	**All (*N* = 71)**	Children* (*N* = 36)	**Adults (*N* = 35)**
**Humoral immune deficiency**	**4**	**2**	**2**
Congenital	3	2	1
Acquired	1	0	1
**Autoimmune inflammatory disorders**	**65**	**34**	**31**
Immune thrombocytopenia	26	17	9
Vasculitis	8	7	1
Myasthenia	8	1	7
Autoimmune neuropathy	7	0	7
Inflammatory myopathy	4	2	2
Guillain-Barré syndrome	3	1	2
Multisystem inflammatory syndrome	4	4	0
Autoimmune neutropenia	2	2	0
Autoimmune hemolytic anemia	1	0	1
Pemphigus	1	0	1
Further conditions	1	0	1
**Antibody-mediated organ rejection**	**2**	**0**	**2**

^*^ ≤ 18 years of age

Ninety percent of the meningitis cases presented ≤ 3 days after initiating immunoglobulin therapy at a dosage of ≤ 4 g and recovered within ≤ 7 days (Table 2). In 22 instances, the authors suggested a link between the onset of meningitis and an overly rapid intravenous infusion of polyclonal immunoglobulin.

**Table 2:** Characteristics of 71 patients 1.0 to 77 years of age with an acute aseptic meningitis temporally associated with an intravenous polyclonal immunoglobulin therapy. Results are presented as frequency (with percentage) or as median and interquartile range

	**All (*N* = 71)**	Children (*N* = 36)*	**Adults (*N* = 35)**	***P* values**
Demographics				
Females to males, *N* (%)	39 (55):32 (45)	17 (47):19 (53)	22 (63):13 (37)	0.2355
Age, years	18 (7.0–34)	7.0 (5.1–10)	35 (26–43)	
Immunoglobulin dose, *N*	65	35	30	
≤ 1 g/kg body weight, *N* (%)	26 (40)	12 (34)	14 (47)	0.2722
2–4 g/kg body weight, *N* (%)	35 (54)	20 (57)	15 (50)	
≥ 5 g/kg body weight, *N* (%)	4 (6.2)	3 (8.6)	1 (3.3)	
Rapid administration, *N* (%)	22 (31)	9 (25)	13 (37)	0.3121
Clinical features				
Increased body temperature, *N* (%)	44 (62)	30 (83)	14 (40)	0.0002
Nausea, vomiting, *N* (%)	49 (69)	25 (69)	24 (69)	> 0.9999
Headache, *N* (%)	61 (86)	31 (86)	30 (86)	> 0.9999
Neck stiffness, *N* (%)	60 (85)	32 (89)	28 (80)	0.3434
Altered level of consciousness, *N* (%)	0	0	0	> 0.9999
Seizures, *N* (%)	0	0	0	> 0.9999
Anaphylaxis, serum sickness, *N* (%)	0	0	0	> 0.9999
Neuroimaging studies performed, *N* (%)	26 (37)	12 (33)	14 (40)	0.6268
Cerebrospinal fluid analysis				
White cell count, × 10^6^/L	328 (103–1324)	487 (150–1486)	277 (80–1150)	0.4675
Predominance (≥ 51%) of neutrophils, *N*	47 (66)	29 (78)	18 (51)	0.3652
Presence of eosinophils, *N*	2	0	2	0.2394
Time, latency, *N*	71	36	35	
≤ 3 days, *N* (%)	62 (87)	30 (83)	32 (91)	0.3654
4–7 days, *N* (%)	7 (9.8)	4 (11)	3 (8.6)	
≥ 8 days, *N* (%)	2 (2.8)	2 (5.6)	0	
Time to recovery, *N*	63	31	32	
≤ 3 days, *N* (%)	43 (68)	29 (94)	14 (44)	< 0.0001
4–7 days, *N* (%)	14 (22)	2 (6.5)	12 (38)	
≥ 8 days, *N* (%)	6 (9.5)	0	6 (19)	
Recurrences, *N*				
1 episode, *N*	7	3	4	
≥ 2 episodes, *N*	1	0	1	
Sequelae, *N*	0	0	0	

^*^ ≤ 18 years of age

The cerebrospinal fluid white cell pleocytosis was predominantly neutrophilic in two-thirds of cases. A cerebrospinal eosinophilia was reported in a small minority of cases. Recurrences after re-exposure to intravenous immunoglobulin were reported in about 10% of cases [12, 13, 20–22, 24, 37, 49]. In at least three cases, recurrences occurred after the administration of a different product [12, 22, 37]. No sequelae were observed.

The body temperature was more frequently increased (*P* = 0.0002), and the time to recovery was shorter (*P* < 0.0001) in children as compared to adults.

Information on the employed polyclonal intravenous immunoglobulin was provided in 41 cases: Sandoglobulin® (*N* = 8), Gamunex® (*N* = 6), Privigen® (*N* = 4), Gamimune® (*N* = 3), Gammagard® (*N* = 3), Biotransfusion® (*N* = 2), Endobulin® (*N* = 2), Flebogamma® (*N* = 2), Intragam® (*N* = 2), Kenketsu Glovenin® (*N* = 2), Kenketsu Venilon® (*N* = 2), Octagam® (*N* = 1), Polygam® (*N* = 1), Polyglobin® (*N* = 1), Tegeline® (*N* = 1), and Venoglobulin® (*N* = 1).

#### Case Series and Pharmacovigilance Studies

**Fig. 2:**
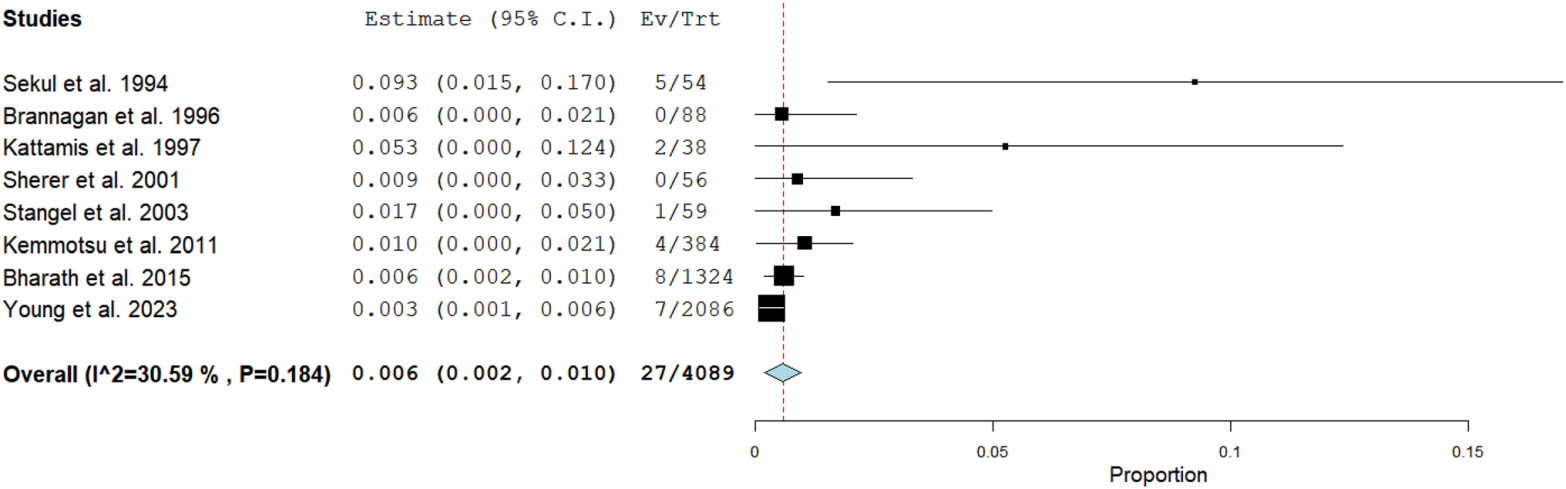
Acute aseptic meningitis temporally associated with intravenous polyclonal immunoglobulin therapy. Proportion meta-analysis with calculation of pooled prevalence of this condition including 95% confidence interval values

Eight retrospective case series [25, 43, 48, 59–63] published between 1994 and 2023 addressed the prevalence of acute aseptic meningitis in a total of 4089 patients treated with a polyclonal intravenous immunoglobulin, as shown in Fig. 2. A pooled prevalence of 0.6% (95% confidence interval, 0.2–1.0%) was noted. No significant heterogeneity was detected (*I*^2^-test, 30.59%), and the Egger’s test did not show significant publication bias (*P* = 0.42).

Furthermore, the pharmacovigilance safety database of Gammagard® [64], a polyclonal immunoglobulin, disclosed a total of 144 episodes of acute meningitis in 136 patients with a male to female ratio of 0.7. For females, the relative frequency of affected patients significantly decreased in a linear fashion with increasing age. Such a tendency was not observed in males.

Finally, the French Pharmacovigilance Database [65] contained 198 cases of drug-induced aseptic meningitis observed between 1985 and 2017: 21 (11%) associated with vaccines, 23 (13%) associated with monoclonal antibodies, 31 (16%) associated with antimicrobials (most frequently amoxicillin or cotrimoxazole), 39 (20%) associated with paracetamol or nonsteroidal anti-inflammatory drugs, and 84 (42%) associated with polyclonal intravenous immunoglobulin. Meningitis was temporally associated with following products: Privigen® (*N* = 31), Tegeline® (*N* = 21), Clairyg® (*N* = 15), Octagam® (*N* = 7), Sandoglobulin® (*N* = 5), and information not provided (*N* = 5).

## Discussion

The results of this systematic review and meta-analysis can be summarized as follows: (1) acute aseptic meningitis temporally associated with high-dose intravenous polyclonal immunoglobulin therapy mostly occurs in individuals, who are affected by an autoimmune disease. The estimated incidence is approximately one case per 200 treatments; (2) nausea, vomiting, headache, neck stiffness, and fever develop within 3 days after receiving a total immunoglobulin dose of 4 g or less. Cerebrospinal fluid analysis reveals an otherwise unexplained white cell pleocytosis, which, in most cases, is predominantly neutrophilic. An eosinophilic pleocytosis is occasionally observed [66]; (3) all cases remit without sequelae within 7 days after discontinuing the polyclonal immunoglobulin, with children experiencing a shorter recovery period compared to adults; and (4) there is no clear relationship with a specific polyclonal immunoglobulin product.

A temporal relationship between polyclonal immunoglobulin therapy and meningitis does not immediately imply causality. Four factors insinuate that the link may be causal. First, the meningitis was otherwise unexplained. Second, the features of meningitis rapidly remitted after discontinuing immunoglobulins without any sequelae. Third, approximately 10% of patients included in this review experienced a recurrence after re-exposure to immunoglobulin therapy. Fourth, a cause-effect relationship is currently admitted for cases of acute aseptic meningitis occurring on treatment with some nonsteroidal anti-inflammatory agents or antimicrobials [7].

Polyclonal immunoglobulin preparations manufactured for intravenous administration are extracted from a large pool of donors and contain purified (95% or more) polyvalent immunoglobulin G [67, 68]. However, there are some differences in manufacturing, and different stabilizers (such as glucose, maltose, or sucrose) are used in the excipients [7, 68]. No relationship was noted between aseptic meningitis induced by polyclonal immunoglobulin therapy and a particular preparation. Furthermore, changing the product did not prevent the condition. It is therefore currently assumed that meningitis is not induced by stabilizers or excipients but directly by the immunoglobulin. The administration of exogenous proteins including polyclonal immunoglobulins is occasionally followed by either an anaphylactic or a serum sickness reaction. An anaphylactic reaction occurs minutes to hours after administration and is characterized by acute onset skin and mucosal lesions together with respiratory, cardiovascular, and intestinal features [69]. A serum sickness reaction occurs 7 to 14 days after exposure and is characterized by fever, skin rash, and joint pain [70]. The patients who developed meningitis after intravenous polyclonal immunoglobulin administration did not exhibit any additional features indicative of an anaphylactic reaction or serum sickness. Meningitis has, at times, been linked to a very rapid polyclonal immunoglobulin administration. Conversely, subcutaneous administration of polyclonal immunoglobulins, which provides more consistent blood levels of immunoglobulin G, is gaining popularity due to a lower likelihood of adverse reactions [4]. For instance, there is only one documented case of aseptic meningitis following subcutaneous immunoglobulin administration [71]. It is therefore tempting to assume that meningitis might result from hyperimmunoglobulinemia induced by high-dose intravenous immunoglobulin therapy.

Meningitis induced by intravenous immunoglobulin is a diagnosis of exclusion. Therefore, it is imperative to rule out infections and further possible causes of symptoms and signs consistent with a meningitis [1, 2, 7]. Given the risk of aseptic meningitis induced by intravenous polyclonal immunoglobulins, careful consideration of their indication is warranted [4]. We speculate that hospitalists who frequently prescribe intravenous immunoglobulins are familiar with this adverse event, suspect the diagnosis on a clinical basis, and make the final diagnosis ex adjuvantibus based on the disappearance of symptoms and signs after discontinuing immunoglobulin or reducing the speed of administration.

A number of measures have been discussed to reduce the otherwise mild tendency to meningitis associated with the administration of high-dose intravenous immunoglobulins: (a) premedication with paracetamol; (b) good hydration; (c) the infusion rate should initially be slow and subsequently increased if well tolerated; and (d) when aseptic meningitis induced by intravenous polyclonal immunoglobulin is diagnosed, the drug should be, whenever possible, discontinued [7, 72]. In this setting, antihistamines are also often prescribed. Corticosteroids have also been frequently recommended but without a clear benefit [7, 72].

This work, which was performed after PROSPERO pre-registration in accordance with the PRISMA guidelines [5], has some limitations. First, we did not incorporate cases, in which the diagnosis was not supported by a lumbar puncture. Second, the quality in reporting and documenting individual cases was somehow heterogeneous. Third, the reported cases do not allow to provide well proven preventive and therapeutic recommendations.

In conclusion, the results of the current review and meta-analysis suggest that the administration of polyclonal intravenous immunoglobulins can lead to an acute aseptic meningitis syndrome [73]. Typically, the clinical features are mild and resolve quickly upon discontinuation of immunoglobulins.

## Data Availability

No datasets were generated or analyzed during the current study.
